# The Relationship Between Vitamin D and Postoperative Atrial Fibrillation: A Prospective Cohort Study

**DOI:** 10.3389/fnut.2022.851005

**Published:** 2022-05-10

**Authors:** Amelie H. Ohlrogge, Jan Brederecke, Francisco M. Ojeda, Simon Pecha, Christin S. Börschel, Lenard Conradi, Vanessa Rimkus, Stefan Blankenberg, Tanja Zeller, Renate B. Schnabel

**Affiliations:** ^1^Department of Cardiology, University Heart and Vascular Center Hamburg, Hamburg, Germany; ^2^German Centre for Cardiovascular Research (DZHK), Partner Site Hamburg/Kiel/Lübeck, Hamburg, Germany; ^3^Department of Cardiovascular Surgery, University Heart and Vascular Center Hamburg, Hamburg, Germany; ^4^University Centre of Cardiovascular Science, University Heart and Vascular Center Hamburg, Hamburg, Germany

**Keywords:** atrial fibrillation, postoperative atrial fibrillation, vitamin D, vitamin D supplementation, 25-hydroxyvitamin D

## Abstract

**Background and Aims:**

The relationship between postoperative atrial fibrillation (POAF) and 25-hydroxyvitamin D [25(OH)D] concentration as well as vitamin D supplementation has been discussed controversially. The relation of pre-operative vitamin D status and POAF remains unclear.

**Methods and Results:**

We analysed the risk of POAF in a prospective, observational cohort study of n = 201 patients undergoing coronary artery bypass graft surgery (CABG) with 25(OH)D concentration. The median age was 66.6 years, 15.4% were women. The median (25th/75th percentile) vitamin D concentration at baseline was 17.7 (12.6/23.7) ng/ml. During follow-up we observed 48 cases of POAF. In age, sex, and creatinine-adjusted analyses, 25(OH)D was associated with an increased risk of POAF, though with borderline statistical significance [odds ratio (OR) 1.85, 95% confidence interval (CI) 0.87–3.92, p-value 0.107], in further risk factor-adjusted analyses the results remained stable (OR 1.99, 95% CI 0.90–4.39, p-value 0.087). The subgroup with vitamin D supplementation at baseline showed an increased risk of POAF (OR 5.03, 95% CI 1.13–22.33, p-value 0.034).

**Conclusion:**

In our contemporary mid-European cohort, higher 25(OH)D concentration did not show a benefit for POAF in CABG patients and may even be harmful, though with borderline statistical significance. Our data are in line with a recent randomised study in community-based adults and call for further research to determine both, the clinical impact of elevated 25(OH)D concentration and vitamin D supplementation as well as the possible underlying pathophysiological mechanisms.

## Graphical Abstract

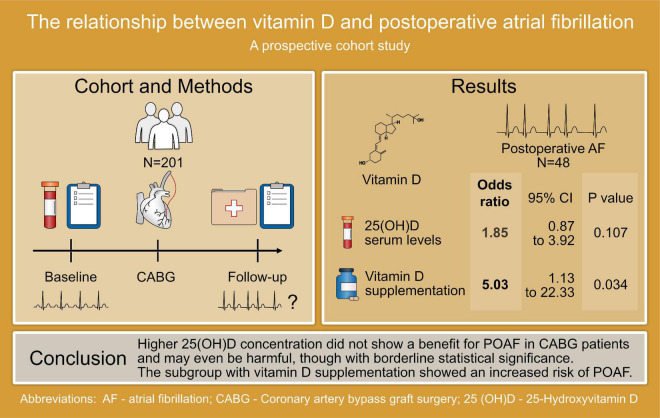

## Introduction

Atrial fibrillation (AF) is a common and serious cardiac arrythmia, with a lifetime risk of 1 in 3 individuals of European ancestry (1, 2). A relevant increase in prevalence is expected due to a longer lifespan and increasing risk factors over the next decades (3, 4). AF is associated with an elevated risk of stroke, heart failure, dementia, and death (5–12). AF often occurs after surgery as postoperative atrial fibrillation (POAF), especially after thoracic and cardiac surgery with incidence rates reported between 20 and 40% for most cardiac surgeries and up to 60% for valve surgery (13–15). The mechanisms of surgery acting as a trigger of AF are not entirely understood. Electrolyte imbalances, inflammation and ischaemia are discussed as triggering factors. POAF increases the length of hospital stay, as well as short- and long-term risk of stroke, mortality and medical costs (16–18). Further, POAF increases the risk of long-term AF about 4–5-fold (17, 19). Understanding the pathophysiology behind the development of AF is key to risk prediction and for potential preventive efforts.

Vitamin D deficiency is a common condition in many societies and influenced, e.g., by geographical latitude and season. Vitamin D status is usually assessed by measuring circulating 25-hydroxyvitamin D [25(OH)D]. Its deficiency is known to be a major risk factor for musculoskeletal diseases; but has also been debated as a risk factor for many other conditions, such as immunologic, endocrine, or cardiovascular. The role of vitamin D and possible benefits of supplementation in AF development has been discussed controversially over the last decade. Whereas smaller, observational studies have shown an association between vitamin D deficiency and the occurrence of AF (20–23), larger cohort studies as well as randomized controlled trials (RCTs) assessing vitamin D supplementation could not confirm these results (24–29). In contrast, the latest RCT in adults in the community, VITAL (Vitamin D and Omega-3) Rhythm Trial, showed a marginally elevated AF risk, though these results did not reach statistical significance in the overall cohort (25).

Therefore, we aimed understand the relationship between circulating 25-hydroxyvitamin D [25(OH)D] and POAF in a cohort study of patients undergoing coronary artery bypass graft surgery (CABG). Further, we analysed the association of vitamin D supplementation and POAF risk.

## Materials and Methods

### Study Cohort

The Atrial Fibrillation in High-Risk Individuals - Biopsy (AFHRI-B) study is an ongoing, prospective, monocentre cohort study designed to improve POAF risk prediction. AFHRI-B is a sub-study of the clinical cohort study (CCS) conducted at the University Heart and Vascular Centre Hamburg (Germany). We included patients 18 years or older undergoing CABG with the support of a heart-lung machine. Individuals who did not have sufficient knowledge of the German language skills to understand the informed consent forms and to participate in the interview were excluded. We focused on AF not related to severe heart valve disease and thus excluded individuals with planned valve surgery or high-grade valvular disease. Participation in the study was voluntary. Written informed consent was obtained from all participants. The conduct of this study was approved by the Local Ethics Committee.

### Data Collection

Baseline data was collected in form of an interview using a detailed questionnaire, including information on pre-existing conditions, medication, family history, lifestyle, and cardiovascular risk factors. Baseline information was supplemented by a review of the electronical medical record. Blood was taken from the patients before surgery and stored at -80°C. In order to collect postoperative data, questionnaires were mailed to the participants and a standardised telephone follow-up interview took place approximately 30 days after study inclusion. In this interview, changes in wellbeing and habits as well as newly diagnosed cardiovascular diseases, including AF, were recorded. For each study participant, all electrocardiograms available in the electronical medical record were analysed by two experienced investigators. In the case of discrepancies in the diagnosis of AF a third cardiologist or electrophysiologist was consulted. Further information on the course of the postoperative treatment were obtained from the discharge report, rehabilitation discharge letters and from the electronical medical record. The primary outcome was newly diagnosed, postoperative AF. 25(OH)D was measured from ethylenediaminetetraacetic acid plasma using the Abbott ARCHITECT i2000 system by a chemiluminescent microparticle immunoassay. After exclusion of 33 patients with AF diagnosed before surgery, *n* = 201 patients remained for our analysis.

### Statistical Analyses

The statistical analyses were performed using R version 4.0.5 and there was no missing data in the sample. A *p*-value of < 0.05 was considered statistically significant. Levels of 25(OH)D as well as creatinine were log-transformed because visual inspection determined the transformed versions to be closer to a normal distribution. Logistic regression models with POAF as the outcome were fitted and corrected for small numbers in categorical predictors using Firth’s correction.

For the current analyses, clinical covariables besides age and sex comprised body mass index (BMI), ever smoker, diabetes, systolic and diastolic blood pressure, hypertension medication, heart failure, and myocardial infarction.

A secondary set of analyses also included the season (summer/winter) in which the blood sample was taken. Summer was defined as lasting from June 1st to November 30th. Vitamin D deficiency was defined as serum levels below 20 ng/ml. Lastly, vitamin D supplementation was included in another set of analyses.

## Results

### Baseline Characteristics

The baseline characteristics are given in Table 1. A total of *N* = 201 patients was included in the analysis. 31 (15.4%) of the included patients were female. The median age was 66.6 years. *N* = 48 patients (23.9%) of patients developed POAF during follow-up. POAF was persisting in 31 (64,6%) of the patients, whereas 17 (35,4%) of the patients had paroxysmal AF. The median (25th/75th percentile) vitamin D concentration was 17.7 (12.6/23.7) ng/ml, *N* = 140 (69.7%) of patients were enrolled in summer. Vitamin D deficiency occurred in 121 (60.2%) patients. A total of *N* = 8 patients (4.0%) took vitamin D supplementation at baseline.

**TABLE 1:** Baseline characteristics by sex and POAF.

	Total Cohort *N* = 201	Women *N* = 31	Men *N* = 170	No POAF *N* = 153	POAF *N* = 48
**Postoperative atrial fibrillation**					
POAF No. (%)	48 (23.9)	8 (25.8)	40 (23.5)	–	–
Persistent POAF No. (%)	31 (64.6)	6 (75.0)	25 (62.5)	–	31 (64.6)
Paroxysmal POAF No. (%)	17 (35.4)	2 (25.0)	15 (37.5)	–	17 (35.4)
**Cardiovascular risk factors**					
Age (years)	66.6 (58.0, 73.1)	68.1 (64.7, 75.5)	65.4 (57.6, 72.7)	64.1 (57.3, 72.9)	69.6 (65.3, 74.9)
BMI (kg/m^2^)	27.7 (24.7, 30.4)	26.0 (23.8, 28.6)	27.8 (24.8, 30.5)	27.7 (24.5, 30.3)	27.7 (25.4, 31.2)
Systolic blood pressure (mm Hg)	133.0 (120.0, 145.0)	135.0 (123.5, 143.0)	133.0 (120.0, 145.0)	133.0 (120.0, 145.0)	130.5 (120.0, 145.2)
Diastolic blood pressure (mm Hg)	77.0 (70.0, 82.0)	75.0 (68.5, 83.0)	77.0 (70.0, 82.0)	78.0 (70.0, 82.0)	73.0 (67.0, 81.5)
Hypertension medication No. (%)	164 (81.6)	28 (90.3)	136 (80.0)	125 (81.7)	39 (81.2)
Ever-smoker No. (%)	147 (73.1)	21 (67.7)	126 (74.1)	111 (72.5)	36 (75.0)
**Pre-existing conditions**					
Heart failure No. (%)	37 (18.4)	6 (19.4)	31 (18.2)	27 (17.6)	10 (20.8)
Myocardial infarction No. (%)	78 (38.8)	10 (32.3)	68 (40.0)	61 (39.9)	17 (35.4)
Diabetes No. (%)	71 (35.3)	10 (32.3)	61 (35.9)	53 (34.6)	18 (37.5)
**Vitamin D status**					
Vitamin D (ng/ml)	17.7 (12.6, 23.7)	12.6 (10.8, 20.5)	17.9 (13.2, 23.8)	16.9 (12.6, 22.7)	20.7 (13.1, 27.2)
Vitamin D deficiency (%)	121 (60.2)	22 (71.0)	99 (58.2)	98 (64.0)	23 (47.9)
Vitamin D supplementation No. (%)	8 (4.0)	2 (6.5)	6 (3.6)	3 (2.0)	5 (10.4)
Creatinine (mg/dl)	0.8 (0.7, 1.0)	0.8 (0.7, 0.8)	0.8 (0.7, 1.0)	0.8 (0.7, 1.0)	0.8 (0.7, 0.9)
Vitamin D measurement in summer No. (%)	140 (69.7)	106 (69.3)	34 (70.8)	106 (69.3)	34 (70.8)

*For continuous variables the median, the 25th, and the 75th percentile are displayed.*

*^1^BMI, body mass index; POAF, postoperative atrial fibrillation.*

Patients, who developed POAF were older, had higher vitamin D concentrations and took vitamin D supplementation more often. They also had higher rates of diabetes or heart failure and were more frequently current or former smokers.

### Correlations Between Biomarkers and Covariates

**FIGURE 1:**
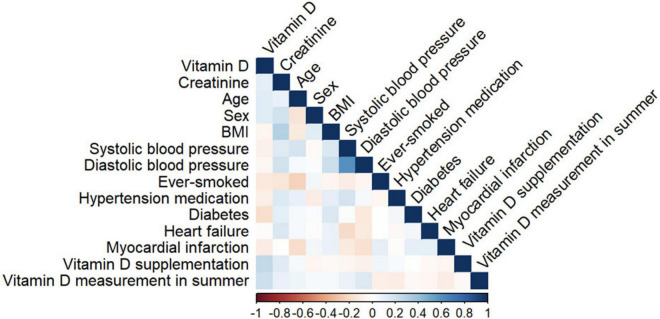
Heat map of correlations of biomarkers and covariates. The colour-coded scale is shown below.

We calculated the correlations between the biomarkers and covariates as shown in Figure 1. Continuous variables are shown as medians [interquartile range; binary variables are shown as counts (frequencies)].

The strongest correlations were observed between 25(OH)D concentration and vitamin D supplementation (*r* = 0.27). 25(OH)D concentration and measurement in summer showed a correlation of 0.22. Weak correlations between vitamin D and the following covariates were observed: creatinine, age, sex, ever smoking, diabetes.

### Logistic Regressions

A first logistic regression model showed higher 25(OH)D concentration to be related to incident AF with borderline statistical significance [odds ratio (OR) 1.85, 95% confidence interval (CI) 0.87–3.92, *p*-value 0.107] when adjusted for age, sex, and creatinine. In analyses further adjusted for clinical variables the results remained stable (OR 1.99, 95% CI 0.90–4.39, *p*-value 0.087).

**FIGURE 2:**
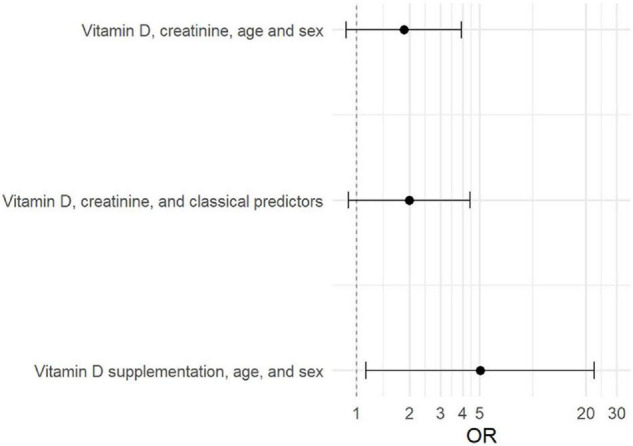
OR-plot showing the odds ratios and 95% confidence intervals for the logistic regression models.

In a secondary analysis, vitamin D supplementation in a model adjusted for age and sex was associated with an increased risk (OR 5.03, 95% CI 1.13 to 22.33, *p*-value 0.034). The ORs and CIs of all models are compared in Figure 2.

## Discussion

In our contemporary cohort we did not observe a benefit for higher 25(OH)D concentration or vitamin D supplementation in relation to POAF. In contrast, elevated 25(OH)D concentration showed an increased risk for the occurrence of POAF, though these results reached only borderline statistical significance. Vitamin D supplementation at baseline was significantly associated with an increased risk of POAF in this subgroup, however, the small size of this subgroup needs to be taken into consideration and these findings must thus be treated with caution.

The effects of vitamin D are pleiotropic and involve the modulation of the renin-angiotensin-aldosterone-system, inflammation, and vascular calcification among others (30–32). Cardiomyocytes express the vitamin D receptor. Alterations of this receptor have genomic effects and change contractility and relaxation of the heart (33, 34). Vitamin D affects K^+^- and Ca^2+^- currents in mouse ventricular myocytes (35, 36). Here, a link to the arrhythmia might be established. Another pathophysiological attempt of explanation focuses on the interaction between vitamin D and electrolytes, in particular calcium, but also phosphate and magnesium through parathyroid hormones. Electrolyte imbalance may be causal in the development of AF. It needs to be considered that most of the published literature describes pathophysiological findings for vitamin D deficiency, not for elevated concentration.

The role of 25(OH)D concentration across the whole range of blood concentration for the development of AF remains unclear. Small case-control studies have shown an association between low vitamin D serum levels and incident AF (20–23), whereas larger cohort studies could not confirm these results (26–29). Two recent, large, prospective, randomised, placebo-controlled studies could not show any benefit of vitamin D supplementation for the occurrence of AF (24, 25). In contrast, the VITAL Rhythm Study, a double-blind, randomised, placebo-controlled study, also showed a tendency for an increased AF risk associated with long-term 2,000 IU of vitamin D_3_ supplementation (25).

Similarly, even though vitamin D deficiency has been shown to be a risk factor for many cardiovascular conditions in multiple observational studies, in many areas large RCTs examining vitamin D supplementation could not demonstrate a convincing benefit of this intervention on hypertension (37), cardiovascular events (38), cardiovascular or all-cause mortality (39). It is possible, that vitamin D deficiency is not causal, but confounded by its association with lifestyle, cardiovascular risk factors, and comorbidities.

Our subgroup results in patients on vitamin D supplementation who showed an increased risk of POAF should be viewed cautiously and in the light of potential confounding-by-indication. These are observational findings. Patients undergoing major cardiac surgery such as CABG often are a particularly vulnerable and frail group which is not directly comparable to the general population. The median age in our cohort was 66.6 years. Many patients had significant comorbidities such as heart failure, prior myocardial infarction, or diabetes mellitus. The perioperative period is marked by physical and psychological stress as well as severe changes in the daily habits like reduced physical and mental activity (40). In addition, there can be alterations in nutrition caused by aspects such as decreased appetite or hospital diet with short-term effects on vitamin D concentrations (41–43). The pre-existing conditions that cause the prescription of vitamin D supplementation in the primary care setting might also increase the risk for AF. Furthermore, patients who take over the counter supplements may significantly differ in lifestyle. On the other hand, including recent evidence from RCTs an actual harmful effect of vitamin D on the development of AF should be considered. Thus, further research on the mechanisms of vitamin in AF is required.

## Limitations

This study is limited by its observational nature which is prone to bias and confounding. The number of outcomes was relatively small, in particular the subgroup of patients with vitamin D supplementation was too small to derive final conclusions. Therefore, the results need to be interpreted with caution. Also, the examined objective was POAF, which in its pathophysiology, patient characteristics as well as clinical presentation is different from incident AF without preceding cardiac surgery. Thus, these results are not entirely transferrable to AF in the general population. However, our findings are in line with recent studies on vitamin D in AF in settings other than POAF. Therefore, further research is necessary to determine both, the clinical impact of 25(OH)D concentration and vitamin D supplementation as well as the potential underlying pathophysiological mechanisms to generate robust evidence on this central vitamin and frequently used supplement. Ideally, clinical trials of vitamin D supplementation could be set up.

## Conclusion

In contrast to previous observational data, higher 25(OH)D concentration and vitamin D supplementation were not beneficial in relation to the risk of POAF in our study of patients undergoing CABG. Elevated 25(OH)D concentration and vitamin D supplementation rather revealed an increased OR for POAF. Whereas our data need confirmation, they also show the need for further research on the role of vitamin D and the serious and costly disease POAF.

## Disclosure

RS has received lecture fees and advisory board fees from BMS/Pfizer outside this work.

## Data Availability Statement

The raw data supporting the conclusions of this article will be made available by the authors, without undue reservation.

## Ethics Statement

The studies involving human participants were reviewed and approved by the Ethikkommission Ärztekammer Hamburg. The patients/participants provided their written informed consent to participate in this study.

## Author Contributions

AO, JB, TZ, and RS: conceptualization. AO, JB, FO, and RS: methodology. JB and FO: formal analysis and writing—original draft preparation. VR, CB, SP, LC, and TZ: resources. VR and RS: data curation. JB, FO, SP, CB, LC, VR, SB, TZ, and RS: writing—review and editing. RS and FO: supervision. RS: project administration and funding acquisition. All authors contributed to the article and approved the submitted version.

## Conflict of Interest

The authors declare that the research was conducted in the absence of any commercial or financial relationships that could be construed as a potential conflict of interest.

## Publisher’s Note

All claims expressed in this article are solely those of the authors and do not necessarily represent those of their affiliated organizations, or those of the publisher, the editors and the reviewers. Any product that may be evaluated in this article, or claim that may be made by its manufacturer, is not guaranteed or endorsed by the publisher.

## Abbreviations

AF: atrial fibrillation
BMI: body mass index
CABG: coronary artery bypass graft surgery
CI: confidence interval
EDTA: ethylenediaminetetraacetic acid
OR: odds ratio
POAF: postoperative atrial fibrillation
RCT: randomised controlled trial
25(OH)D: 25-hydroxyvitamin D.
